# Supplementary material for: The Effect of Fear of Infection and Sufficient Vaccine Reservation Information on Rapid COVID-19 Vaccination in Japan: Evidence From a Retrospective Twitter Analysis
Source: J Med Internet Res. 2022 Jun 9;24(6):e37466. doi: 10.2196/37466 (PMC9186499; doi:10.2196/37466)
Supplement: Multimedia Appendix 1 [file jmir_v24i6e37466_app1.docx]

Table 1. The keywords used for the selection of vaccine-related tweets and the corresponding translation. English keywords were in low-case because all the tweets were low-cased.

| Keywords | Translation | Keywords | Translation |
| --- | --- | --- | --- |
| ワクチン | vaccine | vaccine | - |
| 接種 | inject | pfizer* | - |
| 注射 | inject | ファイザー | Pfizer |
| 打つ | shot | astra, zeneca | - |
| 投与 | inject | アストラ, ゼネカ | Astra, Zeneca |
| mRNA | - | modelna**, moderna | - |
| 副作用, 副反応 | side effect | モデルナ | Moderna |

* Only the vaccine brands approved by the Japanese government were included in the keywords.

** The misspelling of “moderna” was also included in the keywords because we noticed that some Japanese Twitter users tend to write “Modelna” instead of “Moderna” according to the pronuncitation of it in Japanese.

Table 2. English translations used in our paper and the corresponding original Japanese words.

| English translation | Original word | English translation | Original word |
| --- | --- | --- | --- |
| mutation | 変異 | die | 死亡 |
| Pfizer | ファイザー | USA | アメリカ |
| Japan | 日本 | situation | 状況 |
| infect | 感染 | world | 世界 |
| effect | 効果 | above | 以上 |
| AstraZeneca | アストラゼネカ | measures | 対策 |
| information | 情報 | delta | デルタ |
| medical-care | 医療 | India | インド |
| news | ニュース | Tokyo | 東京 |
| reserve | 予約 | venue | 会場 |
| infected | 感染者 | Moderna | モデルナ |
| side effect | 副反応 | available | 可能 |
| severe | 重症 |  |  |

|  |  |  |  |
| --- | --- | --- | --- |


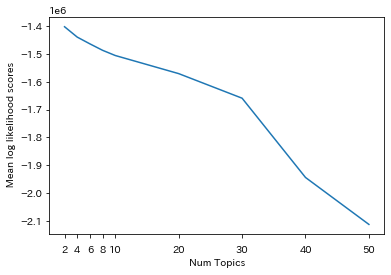


Fig. 1. Mean Log likelihood scores for different LDA topic numbers using five-fold cross validation.
